# Essentials of Operative Dentistry

**Published:** 1911-07-15

**Authors:** 


Essentials of Operative Dentistry.—This is a new manual written by W. Clyde Davis, B. S., M. D., D. D. S., Professor of Operative Dentistry in Lincoln Dental College.

The authors purpose as set forth in his preface is to furnish a text for students, that can be mastered in the shortest time, and at a price that will leave him no excuse for not buying it. At first glance we desire to congratulate the author on his good sense in including in his little work of 266 pages operative dentistry *only*, instead of padding the book out unnecessarily with all the other branches of dentistry. The first chapter is devoted to brief definitions and nomenclature. The definitions are clearly and tersely stated. We note one that is, if not a bit ambiguous, rather involved and difficult to comprehend in its exact meaning; it is as follows: "The median line of the mouth is the anterior border of the antero-posterior perpendicular central plane of the body." The tooth histology, instrument and cavity nomenclature are well, though briefly, set forth in the next three chapters, to be followed by cavity preparation in the next eight chapters. Examinations, alleviation of pain and oral prophylaxis are briefly but clearly stated, and this is followed by a study of the technical procedures, treatment of pulps, root canals, etc. The whole subject of cavity preparation with its nomenclature is well and strongly presented, as is the manipulation of filling materials. The book is not padded in any way with supurfluous descriptions or illustrations, and yet it is not a mere cataloging of principles of practice. It is written in such a good style, and shows such an orderly continuity, that it is delightfully refreshing reading all the way through. It will pay any practitioner to buy and have handy this book for checking up his own method of practice. We suppose it may be had from the dealers, if not from the author who is the publisher.

